# *C. elegans* Expressing Human β_2_-Microglobulin: A Novel Model for Studying the Relationship between the Molecular Assembly and the Toxic Phenotype

**DOI:** 10.1371/journal.pone.0052314

**Published:** 2012-12-21

**Authors:** Luisa Diomede, Cristina Soria, Margherita Romeo, Sofia Giorgetti, Loredana Marchese, Patrizia Palma Mangione, Riccardo Porcari, Irene Zorzoli, Mario Salmona, Vittorio Bellotti, Monica Stoppini

**Affiliations:** 1 Department of Molecular Biochemistry and Pharmacology, Istituto di Ricerche Farmacologiche Mario Negri, Milan, Italy; 2 Department of Molecular Medicine, Institute of Biochemistry, University of Pavia, Pavia, Italy; 3 Centre for Amyloidosis and Acute Phase Proteins, Division of Medicine, Royal Free Campus, University College London, London, United Kingdom; 4 Department of Internal Medicine and Clinical Therapeutics, University of Pavia, Pavia, Italy; Alexander Flemming Biomedical Sciences Research Center, Greece

## Abstract

Availability of living organisms to mimic key step of amyloidogenesis of human protein has become an indispensable tool for our translation approach aiming at filling the deep gap existing between the biophysical and biochemical data obtained *in vitro* and the pathological features observed in patients. Human β_2_-microglobulin (β_2_-m) causes systemic amyloidosis in haemodialysed patients. The structure, misfolding propensity, kinetics of fibrillogenesis and cytotoxicity of this protein, *in vitro*, have been studied more extensively than for any other globular protein. However, no suitable animal model for β_2_-m amyloidosis has been so far reported. We have now established and characterized three new transgenic *C. elegans* strains expressing wild type human β_2_-m and two highly amyloidogenic isoforms: P32G variant and the truncated form ΔN6 lacking of the 6 N-terminal residues. The expression of human β_2_-m affects the larval growth of *C. elegans* and the severity of the damage correlates with the intrinsic propensity to self-aggregate that has been reported in previous *in vitro* studies. We have no evidence of the formation of amyloid deposits in the body-wall muscles of worms. However, we discovered a strict correlation between the pathological phenotype and the presence of oligomeric species recognized by the A11 antibody. The strains expressing human β_2_-m exhibit a locomotory defect quantified with the body bends assay. Here we show that tetracyclines can correct this abnormality confirming that these compounds are able to protect a living organism from the proteotoxicity of human β_2_-m.

## Introduction

*Caenorhabditis elegans* is an extremely versatile and appropriate animal model for mimicking and recapitulating *in vivo* the key molecular mechanisms underlying the gene-and tissue-specific protein misfolding and toxicity related to the human pathogenesis [1]. Despite the evolutionary distance from vertebrates, human proteins substantially maintain their structure and function when they are expressed in *C. elegans* [1]. Many variant proteins associated to human diseases cause a pathological phenotype in worms and this cross-species translation greatly facilitates the study of human diseases in this simple organism. This is particularly true for “gain of function diseases”, including Alzheimer, Parkinson and Huntington diseases, caused by self-aggregation of specific peptides [2]–[4]. Transgenic worms expressing human disease-relevant proteins and peptides also represented a rapid and highly informative system for the screening of putative therapeutic medications at the early stages of drug development with particular regard to aging-related diseases [5]. Alavez et al. [6] have recently shown that *C. elegans* is an excellent biological model for testing compounds that generically counteract the toxicity of protein aggregates. The exposure of nematodes to standard amyloid binding ligands, including thioflavin T and curcumin, has a beneficial effect on the regulators of protein homeostasis and significantly improves the worms lifespan [7].

Although *C. elegans* has been widely employed to investigate a number of neurodegenerative diseases, its application to the study of systemic amyloidoses related to human proteins, i.e. lysozyme, monoclonal light chains, β_2_-microglobulin (β_2_-m) and transthyretin (TTR), is limited. A transgenic *C. elegans* strain expressing wild type human TTR was generated to investigate its putative protective role against the amyloid beta (Aβ) toxicity rather than testing the intrinsic amyloidogenic propensity of TTR [8]. Link and his collaborators showed that the expression of human wild type or Ala60-mutated TTR protected a *C. elegans* transgenic strain from the paralysis induced by Aβ_3–42_ expression but was not associated, *per se*, to the formation of amyloid deposits [8] or to any specific phenotype [9].

*In vivo* models of systemic amyloidosis are urgently needed and this is particularly required for β_2_-m associated disease where any attempt to generate an animal model to recapitulate the key molecular aspects of the pathology have currently failed [10]. β_2_-m is the non-covalently bound light chain of the major histocompatibility complex class I (MHCI). In patients under chronic haemodialysis treatment, it dissociates and converts into amyloid fibrils whose deposition in osteoarticular tissues causes the pathological condition known as dialysis-related amyloidosis (DRA) [11]. Extensive work has been carried out to elucidate the biophysical basis of the propensity of β_2_-m to make amyloid fibrils *in vitro* and identify the specific contribution of a single amino acid residue to the aggregation kinetics. We have shown that, under physiological like conditions, β_2_-m monomers, spontaneously, form oligomeric species which are *on the pathway* of fibril formation [12], [13]. A modest acidification, consistent with the patho-physiologic fluctuations of the pH within the peri-articular tissue, or a small increase of temperature, that can often occur during the haemodialysis treatment, strongly enhanced the protein polymerization [14]. The generation of the first transgenic *C. elegans* strains constitutively expressing the wild type β_2_-m (WT) and two highly amyloidogenic variants, P32G and the truncated form at the 6th N-terminal residue (ΔN6), represent our method to fill the gap existing between the molecular features analyzed *in vitro* and the pathogenic events observed in patients. The P32G variant [15], which was designed to highlight the role of the native cisHis31-Pro32 peptide bond in the fibrillogenesis, enhanced the *in vitro* amyloidogenic potential of wild type protein. ΔN6 is a ubiquitous constituent of β_2_-m amyloid deposits in patients affected by DRA and, due to its capacity to act as a seed in the fibrillogenesis of full length β_2_-m, it could have a crucial role in dictating the clinical history of the disease [16].

*C. elegans* represents a suitable organism to study the tissue damage associated to β_2_-m self aggregation since it lacks the MHCI complex and therefore all the β_2_-m is expressed in a state not bound by the MHCI heavy chain. The abundance of not-bound β_2_-m mimics what occurs during haemodialysis, where circulating free β_2_-m rises 30 to 40 fold [17]. Furthermore, it is worth noting that both collagen, which is structurally similar to the human counterpart, and glycosaminoglycans are highly represented in the basement membrane of the *C. elegans* muscle system [18] and are potent promoters of β_2_-m amyloidogenesis under physiological like conditions [19]. To recapitulate the aggregation process occurring in mammals, we expressed the β_2_-m isoforms in *C. elegans* under the control of a body-wall muscle promoter.

Here we show that both the P32G replacement and ΔN6 truncation remarkably exacerbate the behavioural defects that the expression of wild type human β_2_-m causes in transgenic worms. Mutated and truncated species of β_2_-m had a greater propensity to form *in vivo* soluble oligomeric species than the wild type protein, thus, indicating that the toxicity of these proteins was strictly related to their sequence and aggregation propensity.

To determine whether these new transgenic nematodes might be applied to the screening of compounds that counteract β_2_-m amyloidogenesis and amyloid toxicity, we tested their response to tetracyclines, which have been already reported to inhibit, *in vitro*, the β_2_-m aggregation [20]. These drugs are emerging anti-amyloidogenic compounds and, their ability to counteract the aggregation of various amyloidogenic proteins, including TTR [21], and interact *in vitro* and *in vivo* with Aβ oligomers has been already described [22].

## Materials and Methods

### Construction of *C. elegans* transgenic strains

Transgenic *C. elegans* strains were engineered to express human wild type β_2_-m and two isoforms, P32G and ΔN6, under the control of the body-wall muscle-specific *unc-54* promoter/enhancer. Minigenes encoding wild type β_2_-m and ΔN6 were assembled in two steps. Sequence coding for signal peptide containing compatible cohesive ends (forward sequence: 5′-CTAGCAAAAATGTCTCGCTCCGTGGCCTTAGCTGTGCTCGCGCTACTCTCTCTTTCTGGCCTGGAGGCTGGTAC-3′; reverse sequence: 5′-CAGCCTCCAGGCCAGAAAGAGAGAGTAGCGCGAGCACAGCTAAGGCCACGGAGCGAGACATTTTTG-3′) was inserted between the unique *Nhe*I and *Kpn*I sites of pPD30.38 vector (Addgene) [5]. Subsequently, wild type β_2_-m and ΔN6 sequences (obtained from the plasmids pHN1 and pET11a, respectively) were amplified by using β_2_-m cDNA as template and the oligonucleotide primers 5′-GGGGGTACCATCCAGCGTACTCCAAAG-3′ for the full length, 5′-GGGGGTACCATTCAGGTTTACTCACGTC-3′ for the truncated species and, 3′-CCCGAGCTCTTACATGTCTCGATCCCAC-5′ for both species. The amplified DNA was inserted between the unique *Kpn*I and *Sac*I sites of pPD30.38 previously engineered with the signal peptide. To obtain P32G β_2_-m plasmid, a site-directed mutagenesis of wild type β_2_-m engineered plasmid pPD30.38 was performed, using the following primers: 5′-CTATGTGTCTGGGTTTCATGGATCCGACATTGAAGTTGAC-3′ and 5′-GTCAACTTCAATGTCGGATCCATGAAACCCAGACACATAG-3′. A pPD30.38 plasmid containing only the signal peptide was created as control. DNA sequencing was carried out to confirm that all subcloned plasmids were correct. Transgenes were introduced into MT309 multivulva *C. elegans* strain (Caenorhabditis Genetics Center, CGC, University of Minnesota, USA) by gonad microinjection of a DNA solution containing 25 ng/µl of the β_2_-m construct together with 20 ng/µl of *ttx-3::rfp* and 30 ng/µl of *plin-15(+)* as marker plasmids. Multiple extra-chromosomal lines were established based on both the fluorescent marker and the disappearance of the multivulva phenotype. The transgenic worms maintain the injected DNA as an extra-chromosomal multiply array of variable mitotic and meiotic stability. The transmitting lines established for this study have 70–80% meiotic stability. Thus transgenic worms produce both transgenic and non-transgenic progeny.

Worms were maintained and propagated at 20°C on solid Nematode Growth Medium (NGM) seeded with OP50 *E. coli* (Caenorhabditis Genetics Center, University of Minnesota, USA) as food. To prepare age-synchronized animals, nematodes were transferred to fresh NGM plates after reaching maturity at 3 days of age and allowed to lay eggs overnight. Isolated hatchlings from the synchronized eggs (day 1) were cultured on fresh NMG plates at 20°C.

### Genotype characterization

RNA from adult transgenic worms was prepared using the RNeasy Mini kit (QIAzol Lysis Reagent, Qiagen) and quantified using the NanoDrop apparatus (ThermoScientific). Total RNA was reverse transcribed into cDNA with random primers (Random Hexamers, Applied Biosystems) and the GeneAmp RNA PCR Core kit (Applied Biosystems). A quantitative real-time PCR was performed with Mx3000P QPCR System (Stratagene) using the SYBR Green gene expression assay (Applied Biosystems, AB). Relative quantification of mRNA level was determined using two endogenous standard gene controls, i.e. peripheral myelin gene PMP-22/GAS-3 and cell division cycle 42 (cdc-42, GTP binding protein) according to Hoogewijs [23] and, data analysis was performed with MxPro QPCR Software (Stratagene). All measurements were determined in triplicate. Data points collected correspond to the number of PCR cycles (Ct value) required for the fluorescent signal to cross the detection threshold of the thermal cycler. Ct values were normalized to correct for minor differences in cDNA concentrations by subtracting the average of the Ct values of the reactions in triplicate of each transgenic strain from the geometric mean of Ct values of cdc-42 reactions, and analyzed using the comparative 2^−ΔΔCt^ method [24].

### β_2_-m expression

Transgenic worms were collected with M9 buffer, transferred to tubes, centrifuged and washed twice to remove bacteria. The pellet containing worms was resuspended in lysis buffer (25 mM Tris, pH 7.5, containing 5 mM NaCl, 5 mM EDTA, 1 mM dithiothreitol, DTT, and protease inhibitor mixture) and homogenized by sonication. For dot-blot analysis, equal amounts of proteins from homogenized samples (5 µg) were spotted onto nitrocellulose membranes (Millipore), blocked with 5 mM phosphate buffered solution, pH7.4 containing 0.1% Tween 20 (PBS-T) and incubated overnight with a rabbit polyclonal anti-human β_2_-m antibody (1∶1000 dilution, Dako) or a rabbit polyclonal antibody recognizing high molecular weight oligomers (A11, 1∶1000 dilution, Biosource, USA). Anti-rabbit IgG peroxidase conjugate (1∶10000 dilution, Sigma) was used as secondary antibody. Immunoreactive bands were detected by ECL chemioluminescence (Millipore) and quantified with Quantity One Image Software (Biorad). Data are expressed as density/µg of protein. The β_2_-m species in transgenic populations were identified by western blotting [22]. Equal amounts of protein lysates were filtered using a 30K cut off filter device (Millipore) and, flow through samples were loaded onto gradient 8–18% Excel SDS gel (GE Healthcare) for electrophoresis performed under reducing conditions. Proteins were transferred to Immobilon P membranes and blot was developed with a rabbit polyclonal anti human β_2_-m antibody (1∶1000 dilution, Dako) and anti-rabbit IgG peroxidase conjugate (1∶10000 dilution, Sigma) as primary and secondary antibody respectively. Chemioluminescent substrate was used as above.

### Immunofluorescence

Fluorescence microscopy analysis was carried out on whole worms [25], [26]. Briefly, egg-synchronized L4/young adult worms were collected, rinsed and fixed in 2% p-formaldehyde solution. Fixed worms were subjected to thermal shock and washed twice in 100 mM Tris-HCl solution pH7.4, containing 1% (v/v) Triton X-100 and 1 mM EDTA. Samples were reduced with 2 hours incubation, 37°C, using the same buffer containing 1% β-mercaptoethanol followed by further 15 min incubation, in 25 mM H_3_BO_3_ solution, pH9.2, containing 10 mM DTT, at room temperature. Subsequent steps included: incubation in 25 mM H_3_BO_3_, pH 9.2, containing 1% H_2_O_2_, room temperature for 15 min; extensive washing in 5 mM PBS pH7.4, containing 1% bovine serum albumin, 0.5% Triton X-100, 0.05% sodium azide and, 1 mM EDTA; overnight incubation with the rabbit polyclonal anti- human β_2_-m antibody (1∶100 dilution, Dako), 4°C; extensive washing as above; overnight incubation with an IgG Alexa Fluor 546 goat anti-rabbit antibody (1∶200 dilution, Invitrogen), 4°C. Samples were then mounted on slides for microscopy and observed with an inverted fluorescent microscope (IX-71 Olympus) equipped with a CDD camera (F- VEWII) and images captured.

### Larval growth

One hundred synchronized eggs were plated on fresh NMG plates seeded with OP50, left at 20°C and the number of worms at L1/L2, L2/L3 and L4/adult larval stage were scored after 13, 22 and 40 hours, respectively.

### Life-span

Gravid worms were allowed to lay eggs for 3–4 hours at 20°C, to produce an age-synchronized population. Once the worms reached their reproductive maturity, they were transferred daily until the cessation of egg lying to avoid overlapping generations. Nematodes were transferred every day and their viability monitored until all the worms were scored as dead when they failed to display touch-provoked movement.

### Body Bends assay

Body Bends assays were performed at room temperature using a stereomicroscope (M165 FC Leica) equipped with a digital camera (Leica DFC425C and SW Kit). L3–L4 worms were picked and transferred into a 96-well microtiter plate containing 100 µl of ddH_2_O. The number of left-right movements in a minute was recorded.

To determine the effect of tetracycline in preventing the locomotory defect caused by β_2_-m expression, egg-synchronized transgenic worms (100worms/plate) were placed into fresh NMG plates, 20°C and, seeded with tetracycline-resistant *E. coli* [22]. After thirty-six hours, at their L3/L4 larval stage, worms were fed with 50–100 µM tetracycline hydrochloride or doxycycline (100 µl/plate) and body bends in liquid were scored after 24 hours. Tetracycline hydrochloride and doxycycline were from Fluka (Switzerland) and were freshly dissolved in water before use.

### Pharyngeal pumping assay

Individual L3/L4 transgenic worms were placed into NMG plates seeded with *E. coli* and the pumping behaviour was scored by counting the number of times the terminal bulb of the pharynx contracted over a 1-minute interval.

### Superoxide production

Superoxide anions, in synchronized L3/L4 worms, were estimated using the colorimetric nitro blue tetrazolium (NBT) assay [27]. Superoxide anions were measured in 100 µl sample volume added with 1.5 µl of 50 nM phorbol myristate acetate, 50 µl of 1.8 mM NBT (Sigma-Aldrich, St Louis, MO, USA) and, incubated at 37°C for 30 min. Absorbance was read at 560 nm against blank samples without worm homogenate (Infinite M200 multifunctional micro-plate reader, Tecan, Austria). Superoxide production was expressed as percentage of NBT (absorbance/mg of protein) compared to untreated control worms. The protein content was determined using Bio-Rad Protein assay (Bio-Rad Laboratories GmbH, Munchen, Germany).

### Fluorescent staining of amyloid

Age-synchronized transgenic worms were fixed in 4% paraformaldehyde/PBS pH7.4 for 24 hours at 4°C. Nematodes were stained with 1 mM 1,4-bis(3-carboxy-hydroxy-phenylethenyl)-benzene (X-34) in 10 mM Tris-HCl, pH 8.0 for 4 hours at room temperature [8], destained, mounted on slides for microscopy and observed with inverted fluorescent microscope (IX-71 Olympus); images were acquired with a CDD camera.

### Statistical analysis

Data were analyzed using independent Student's t-test and One-way ANOVA test with GraphPad Prism 4.0 software (CA, USA). A p value<0.05 was considered statistically significant.

## Results

We generated three new transgenic *C. elegans* strains expressing human wild type β_2_-m and two highly amyloidogenic variants and used these novel animal models to elucidate the putative correlation between the aggregation of β_2_-m and its *in vivo* proteotoxicity.

**Figure 1:**
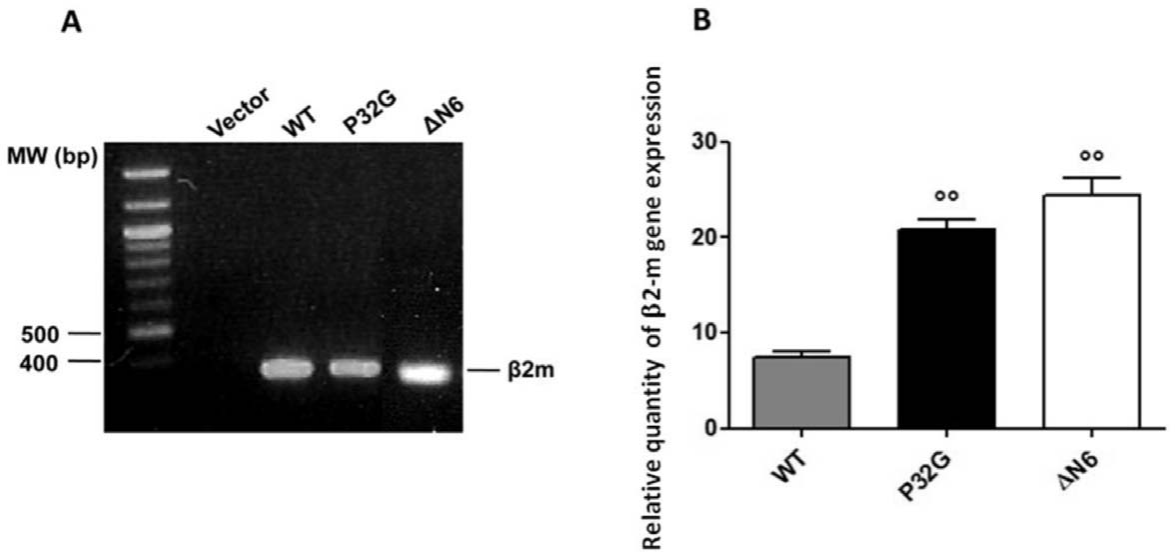
Genotype of *C. elegans* transgenic strains. (**A**) PCR genotyping of adult transgenic worms transfected with the empty vector (vector) or vectors for expression of wild type β_2_-m (WT), P32G or 7–99 truncated form (ΔN6). The expected size of PCR products (about 360 bp) was observed. (**B**) Human β_2_-m mRNA expression in different transgenic strains was normalized to worm cell division cycle 42 (cdc-42, GTP binding protein) as endogenous reference. Data are expressed as mean ± SD of three independent experiments.

Reverse transcription of total RNA, followed by PCR and sequence analysis of the resulting cDNA, confirmed the exact genotype of the three transgenic strains. PCR products, from all the nematode strains, show the expected size of 360 bp (Figure 1A). The relative quantity of human β_2_-m mRNA, normalized to worm cdc-42 content, was significantly higher in both P32G and ΔN6 expressing worms than in WT strain (p<0.01, one-way ANOVA) (Figure 1B).

**Figure 2:**
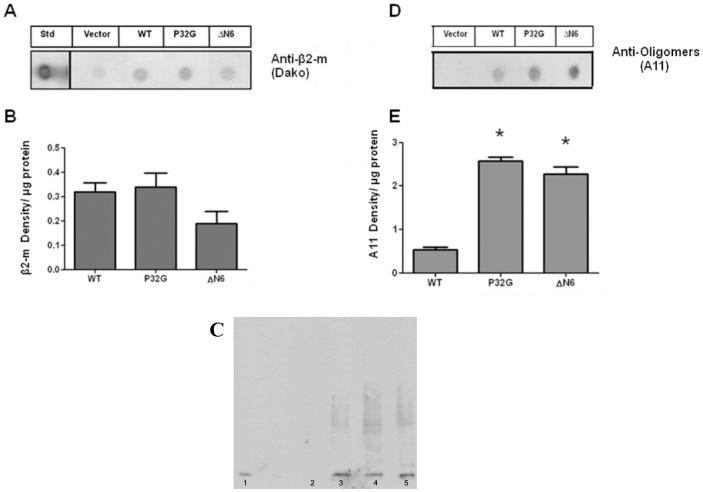
Human β_2_-m protein expression. (**A**) Representative dot blot of β_2_-m (polyclonal anti-human β_2_-m antibody) in transgenic worms and (**B**) quantification of β_2_-m immunoreactive bands. Data are mean values of density of immunoreactive bands/µg of protein ± SE of three independent experiments (N = 6). (**C**) Representative western blot of β_2_-m in control worms (vector), wild type β_2_-m expressing worms (WT), and in nematodes expressing P32G (P32G) or ΔN6 β_2_-m isoform (ΔN6). Day 1 adult worms were collected, processed as described in Methods section, and equal amounts of proteins (40 µg) were loaded on each lane and immunoblotted with polyclonal anti-human β_2_-m antibody (Dako). (**D**) Representative dot blot developed by antibody recognizing oligomers (A11) in transgenic worms and (**E**) quantification of A11-immunoreactive bands. Data are expressed as mean of density of A11 immunoreactive bands/µg of protein ± SE of three independent experiments (N = 9); *p<0.01 vs WT, according to one-way ANOVA.

To correlate the mRNA level with the amount of β_2_-m expressed in the different transgenic strains, worm lysates were analyzed by dot blotting using polyclonal anti-human β_2_-m antibody. Although a faint unspecific band was detected in worms transfected with the empty vector, an increase in β_2_-m related signal was observed in WT, P32G and ΔN6 expressing nematodes as shown in Figure 2A. Quantification of the immunoreactive dots indicated that both WT and P32G transgenic strains expressed comparable β_2_-m levels (0.32±0.04 and 0.34±0.06 density/µg protein for WT and P32G, respectively) whereas a lower, but not statistically significant, protein content was detected in ΔN6 animals (0.19±0.05 density/µg protein) (Figure 2B). SDS-PAGE and Western blot immunostained with polyclonal anti human β_2_-m antibody confirms the relative abundance of the three isoforms (Figure 2C).

The ratio between the relative amount of mRNA and dot-blot immunoreactive β_2_-m signals of the three variants is 26, 67 and 125 for WT, P32G and ΔN6, respectively. These findings suggest that the higher transcription level of P32G and ΔN6 cDNA compared to WT does not result in different β_2_-m protein concentration among the three strains. The dissociation between the mRNA transcription and the protein level is consistent with a putative role of the quality control system in removing the misfolded conformers that are particularly abundant in the case of the two highly amyloidogenic species. The western blot in Figure 2C shows the presence of a monomeric β_2_-m band in the lysates and a smear of aggregated protein that, despite extensive centrifugation and filtration is particularly evident in P32G and ΔN6 samples. Such a feature is also consistent with the well-established propensity of these β_2_-m isoforms to misfold and self-aggregate [15], [16].

The ability of the three β_2_-m isoforms to form oligomeric structures *in vivo* was then explored by performing dot-blot analysis on lysates of worms using the A11 antibody that specifically recognizes the amyloid oligomers. The expression of wild type protein was accompanied by a small A11-positive signal, which became stronger in transgenic worms expressing the two variants (Figure 2D). The quantification of the A11-immunoreactivity indicated that the oligomerization significantly increased 4.8 and 4.3 fold in P32G and ΔN6 mutants, respectively, compared to WT (Figure 2E, p<0.01 vs. WT, one-way ANOVA).

**Figure 3:**
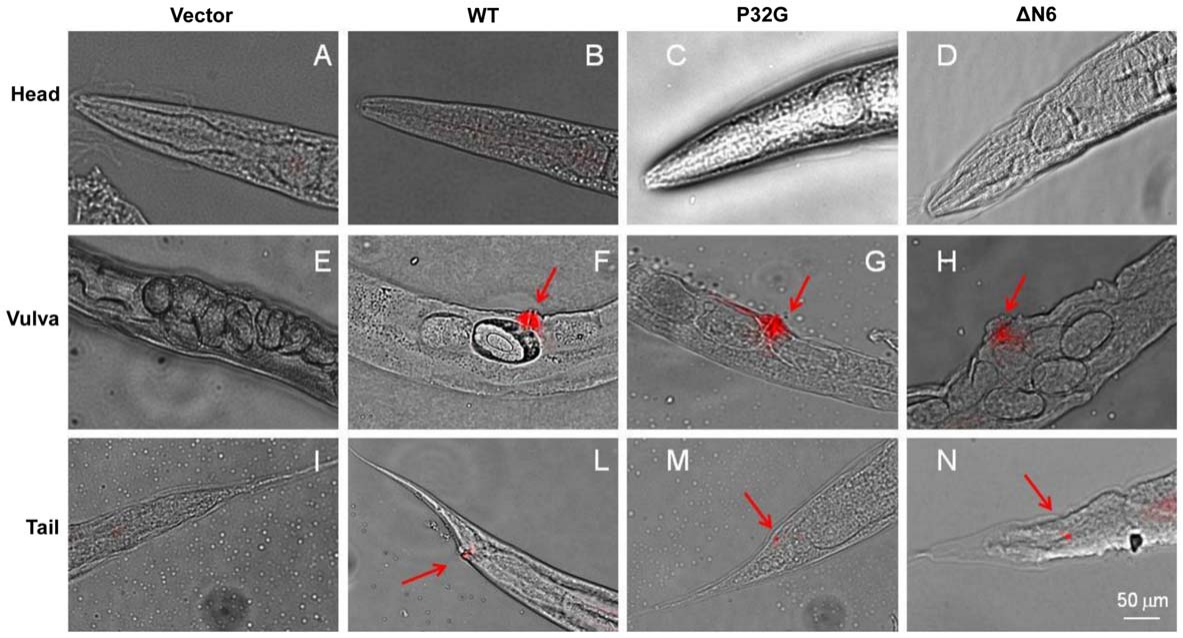
Localization of β_2_-m in transgenic *C. elegans* strains. Overlay of bright field and immunofluorescence images of head, vulva and tail of transgenic *C. elegans* strains. All animals depicted are 2 days adult worms. A specific β_2_-m related signal (red, using a polyclonal anti human β_2_-m antibody) was observed at the vulva muscles and anal sphincter muscle in the tail (red arrows) whereas no signal was observed in the head muscles. Scale bar, 50 µm.

Immunofluorescence studies were carried out to visualize the β_2_-m in transgenic *C. elegans* strains. A β_2_-m-positive signal was observed in the vulva muscles and anal sphincter muscle in the tail regions: it begun at larval stages of WT, P32G and ΔN6 animals (data not shown) and became maximal at day 1-adult age (Figure 3). No signal was detected in worms that were transfected either with the empty vector or alternatively in the head (data not shown). The constitutive expression of the wild type or variant β_2_-m did not lead to the formation of amyloid fibrils, since no X-34 reactive deposits were detected in the vulva and tail muscles of 2 days-old transgenic worms (Figure S1).

**Figure 4:**
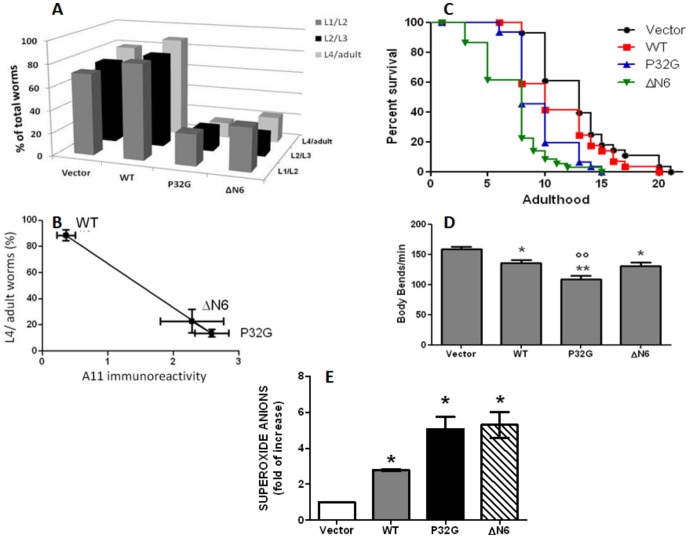
Behavioural phenotypes of transgenic *C. elegans* strains. (**A**) Larval growth of control worms (Vector), wild type β_2_-m expressing worms (WT) and nematodes expressing P32G or 7–99 truncated form of β_2_-m (ΔN6). One hundred synchronized eggs were placed into fresh NMG plates seeded with OP50 as food, and the number of L1/L2, L2/L3 and L4/adult worms were scored after 24, 48 and 72 hours, respectively. Data are expressed as percentage of total worms in the plate at each time point and are given as mean of three independent experiments (N = 300). (**B**) Correlation between oligomers of β_2_-m and reduction in growth rate of transgenic *C. elegans* strains. Percentage of adult worms of each transgenic strain, scored 72 after egg synchronization, was correlated to the the amount of A11-positive oligomeric assemblies detected by dot blotting. Data of both graphic axes represent mean of three independent experiments. (**C**) Kaplan-Meier survival curves of transgenic hermaphrodite adult nematodes. Animals were placed in plates seeded with OP50 starting from L4, cultured at 20°C and transferred to fresh plates for each consecutive other days. Survival rate was scored every day and expressed as percent of survival. Plots are representative of three independent experiments (N = 30). (**D**) Body bends in liquid of transgenic worms. At least three independent assays were performed (N = 100 animals for each group). Data are given as mean of number of body bends/min ± SE, *p<0.05 and **p<0.01 vs. the vector, °°p<0.01 vs. WT, according to one-way ANOVA. (**E**) Superoxide anions production in control worms (Vector), wild type β_2_-m expressing worms (WT) and in nematodes expressing P32G or 7–99 truncated form of β_2_-m (ΔN6). Age-synchronized worms were collected in PBS containing 1.6 ml of 1% Tween 20 and colorimetric NBT assay was carried out as described in Materials and Methods. Results show the fold increase in superoxide production calculated as NBT absorbance/mg of proteins (% NBT) compared to Vector; *p<0.05 vs. vehicle and ° p<0.05 vs. WT, according to one-way ANOVA. Error bars indicate SD.

We also investigated whether the expression of the different isoforms of human β_2_-m resulted in specific toxic behavioural phenotypes. First of all, the effect on the larval growth was considered. Larval growth in *C. elegans* is known to be exponential; therefore the growth rate is constant within larval phases and, reached a plateau in late adulthood [28]. After synchronization, the numbers of worms were scored after 24, 48 and 72 hours that correspond to the L1/L2, L2/L3 and L4/adult larval stages, respectively. WT nematodes exhibited a constant number of worms and a constant growth rate similarly to that observed in animals transfected with the empty vector (Figure 4A). In P32G and ΔN6 transgenic *C. elegans* strains, the percentage of worms reaching the L1/L2 stage was significantly reduced than in WT (83.3% for WT and 27.6% and 37.8% for P32G and ΔN6, respectively, p<0.01 vs. WT, one-way ANOVA). The irregular growth rate compared to WT was also observed at the L2/L3 larval stage (81.4% for WT and 20.0% and 18.7% P32G and ΔN6, respectively, p<0.01 vs. WT, one-way ANOVA, Figure 4A). This resulted in a significant reduction in the percentage of worms reaching the adulthood, being the 88.6% for WT nematodes and 13.8% and 22.9% for P32G and ΔN6 transgenic animals, respectively (p<0.01vs. WT, One-way ANOVA) and indicates that the expression of the mutated or truncated isoforms of the protein affected the nematodes growth and development.

The phenotypic abnormality well correlated with the aggregation pathway of β_2_-m. In particular, a correlation coefficient of R = 0.979 was determined when the percentage of transgenic worms reaching the adulthood, 72 hours after synchronization, was plotted with the amount of A11-positive oligomeric assemblies detected by dot blotting (Figure 4B).

To determine whether β_2_-m affected the health of nematodes and their lifespan, the overall nematodes survival was evaluated. The expression of wild type β_2_-m significantly decreased the median lifespan of transgenic worms compared to nematodes injected with the empty vector (Figure 4C, median survival respectively: 13 days and 10 days for Vector and WT, p<0.05, Wilcoxon test). The insertion of both the P32G mutated gene and deleted ΔN6 sequence similarly shortened the survival of worms by 38% compared to the empty vector (median of survival: 8 days for both P32G and ΔN6, p<0.001 vs. Vector, Wilcoxon test) and by 20% compared to WT (p<0.01, Wilcoxon test). Thus, nematodes expressing the mutated or truncated gene had a shorter lifespan, indicating that, *in vivo*, P32G and ΔN6 show a greater proteotoxicity than WT β_2_-m.

The presence of misfolded proteins in body wall muscle cells can induce dysfunctions in the coordination and motility of *C. elegans* [6].

We investigated whether the presence of β_2_-m in vulva muscles affected the locomotion. It is well known that, in the vulva, hermaphrodite-specific motor neurons make extensive neuromuscular junctions with the vulva muscles affecting the coordination of egg-laying and locomotion (http://www.wormbook.org/chapters/www_egglaying/egglaying.html). The locomotion activity in liquid of β_2_-m expressing worms was then evaluated by quantifying their body bends. Worms transfected with the empty vector had a motility similar to ancestral N2 animals (vector, 158.6±23 body bends/min, N2, 170.3±15, N = 70) indicating that insertion of the transgene without β2-m construct did not affect locomotion. A significant reduction of the body bends, compared to the empty vector, was observed in both WT animals and in worms expressing the two β_2_-m variants. In particular, we observed a significant decrease of the number of body bends per minute by 15% and 18% in WT and ΔN6 expressing strains, respectively. Nematodes expressing P32G mutated gene had a worse motility than WT and ΔN6 animals (p<0.01, one-way ANOVA) with a 32% reduction in body bends compared to worms transfected with the empty vector (Figure 4D).

Oxidative stress is known to occur in transgenic *C. elegans* strains expressing amyloidogenic proteins [29], [30]. We determined superoxide production in β_2_-m expressing worms at L3/L4 larval stage. Superoxide levels rose significantly in all β_2_-m expressing transgenic strains compared to worms transfected with the empty vector (Figure 4E). In addition, nematodes expressing the two β_2_-m variants, ΔN6 and P32G, generated more oxygen free radicals compared to WT indicating that β_2_-m isoforms affect the superoxide production (Figure 4E).

**Figure 5:**
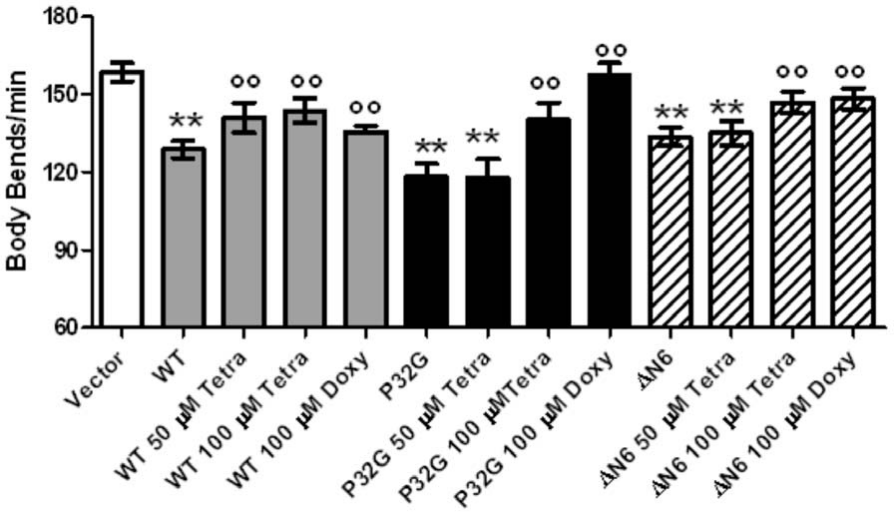
Effect of tetracycline on β_2_-m induced locomotory defect in transgenic *C. elegans* strains. Egg-synchronized control worms (vector), wild type β_2_-m expressing worms (WT), P32G-mutated β_2_-m and ΔN6-truncated β_2_-m expressing nematodes (ΔN6) were placed at 20°C into fresh NMG plates seeded with tetracycline-resistant *E. coli*. At their L3/L4 larval stage, animals were fed with 50–100 µM tetracycline hydrochloride or 100 µM doxycycline (100 µl/plate). Body bends in liquid were scored after 24 hours. At least three independent assays were performed. Data are mean of number of body bends/min ± SD; **p<0.01 vs. the Vector, °°p<0.01 vs. the respective untreated group, according to one-way ANOVA (N = 60 animals for each group).

To determine whether the new transgenic nematodes can be used for testing *in vivo* the pharmacological effect of compounds inhibiting amyloidogenesis and amyloid toxicity [22], we investigated the ability of tetracyclines to counteract β_2_-m proteotoxicity *in vivo*. Worms were fed with either vehicle or 50–100 µM tetracycline hydrochloride for 24 hours and body bends were scored. As shown in Figure 5, 50 µM tetracycline completely abolished the body bends reduction caused by WT β_2_-m expression in worms, whereas it resulted ineffective in P32G and ΔN6 nematodes. A higher dose of 100 µM tetracycline was required to recover the locomotory defect in transgenic *C. elegans* strains expressing the two variants. The number of body bends of worms transfected with the empty vector was not affected by tetracycline administration (data not shown). Similar effects were observed after feeding worms with doxycycline, another tetracycline-derived compound that was shown to be effective *in vitro* against the β_2_-m aggregation and cytotoxicity (Figure 5) [20].

## Discussion

We report the first model of transgenic *C. elegans* expressing and directing human β_2_-m in the muscular system. The comparative analysis of the phenotype of strains expressing the wild type protein and two highly amyloidogenic isoforms of β_2_-m suggests that protein misfolding and aggregation propensity, that were previously observed *in vitro* [15], [16], are confirmed *in vivo* using this complex living organism.

Although we have not found genuine amyloid fibrils in the worms, the strains expressing P32G and ΔN6 generate a higher amount of oligomeric species that are generally considered the toxic species of amyloid aggregates. The ratio between the amount of β_2_-m expressed in each *C. elegans* transgenic strain and the level of mRNA (protein/mRNA) suggests that, when the mutated and truncated forms of β_2_-m are produced, the worms activate a degradative response toward the more amyloidogenic species. This is particularly informative for the truncated form of β_2_-m (ΔN6) that is ubiquitously present in all the amyloid deposits of patients affected by DRA [31] where ΔN6 is considered a strong promoter of amyloidogenesis of wild type β_2_-m [32]. Its susceptibility to proteolytic degradation is well documented by studies of limited proteolysis [33] and is consistent with the evidence that, in DRA patients, the ΔN6 is confined to the amyloid fibrils where is protected from proteolytic degradation, but it is undetectable in circulating blood [31]. Even thought the amount of ΔN6 escaping the quality control machinery is probably lower than that of wild type β_2_-m, the kinetics of ΔN6 self-aggregation in *C. elegans* is so fast and efficient that a population of cytotoxic oligomeric β_2_-m is nonetheless formed. Data regarding the P32G variant can be similarly interpreted, although we cannot assume any clinical-pathologic correlation in humans because it only represents a protein model.

It is worth of note that the expression of wild type full-length β_2_-m, *per se*, affects the physiology of the worm, but the expression of the two more amyloidogenic species highly enhanced the damage to the biological cycle of the worms. The harm caused by β_2_-m might depend on the aggregated species, as demonstrated by the statistically significant inverse correlation that we observed between the concentration of oligomers and larval growth (Figure 4B). A crucial role on larval development is played by mitochondrial efficiency [34], and mitochondria represent sensitive target of the cytotoxic amyloid aggregates generated by several amyloidogenic peptides [35] and proteins [36]. The increased concentration of the reactive oxygen species, produced in all the *C. elegans* strains and, particularly in those expressing the P32G and ΔN6 β_2_-m (Figure 4E), is perfectly consistent with the involvement of the mitochondrial function in the mechanism of toxicity.

In addition to abnormalities of the biological cycle, worms expressing β_2_-m, display significant defects in locomotory function documented through the analysis of the frequency of body bends. This abnormality is reported in other *C. elegans* strains that express other fibrillogenic polypeptides including Aβ protein, synuclein and huntingtin [2], [37] and, therefore, the damage observed in the β_2_-m transgenes might be common to other amyloidogenic proteins in their oligomeric state. Deposition of protein aggregates in the vulva and the tail, as it occurs in our transgenes, can severely affect the locomotion of worms [38], however we cannot exclude that soluble β_2_-m oligomers could cause *per se* a systemic cytotoxicity thus damaging the efficiency of muscles not directly targeted by deposition of protein aggregates.

We are aware that this model is susceptible to several improvements and variations such as the expression of β_2_-m in other organs than muscles, but currently it represents the only available system of expression of human β_2_-m in a living organism. It can also be used for studying other isoforms of β_2_-m, including the first amyloidogenic variant of β_2_-m which causes a systemic amyloidosis unrelated to the haemodialytic procedure [39]. Nevertheless animal models of β_2_-m related amyloidosis are essential to discover and validate new effective drugs. The capacity of tetracyclines to abrogate the locomotory abnormalities caused by β_2_-m expression is remarkable and, indicate that the *C. elegans* strains can be considered for testing, in living complex organisms, the pre-clinical efficacy of molecules, whose capacity of inhibiting fibrillogenesis and cytotoxicity of β_2_-m, have been tested only with isolated proteins and cell cultures [20], [40].

## Supporting Information

Figure S1 **X-34 staining of whole transgenic worms.** Representative images of X-34 staining of whole-mount and fixed sections of WT and P32G transgenic worms. Animals depicted are 1–2 day adult worms. X-34 staining was visualized at short wavelength excitation. Red arrows pointed at vulva muscles and anal sphincter muscle in the tail where a specific β_2_-m related signal was observed with immunofluorescence studies (see Figure 3). The X-34 signal observed was not due to amyloid deposition but to intestine related non-specific background. Scale bar, 20 µm.
(TIF)
