# Supplementary figures and images for: C. elegans Expressing Human β2-Microglobulin: A Novel Model for Studying the Relationship between the Molecular Assembly and the Toxic Phenotype
Source: PLoS One. 2012 Dec 21;7(12):e52314. doi: 10.1371/journal.pone.0052314 (PMC3528749; doi:10.1371/journal.pone.0052314)

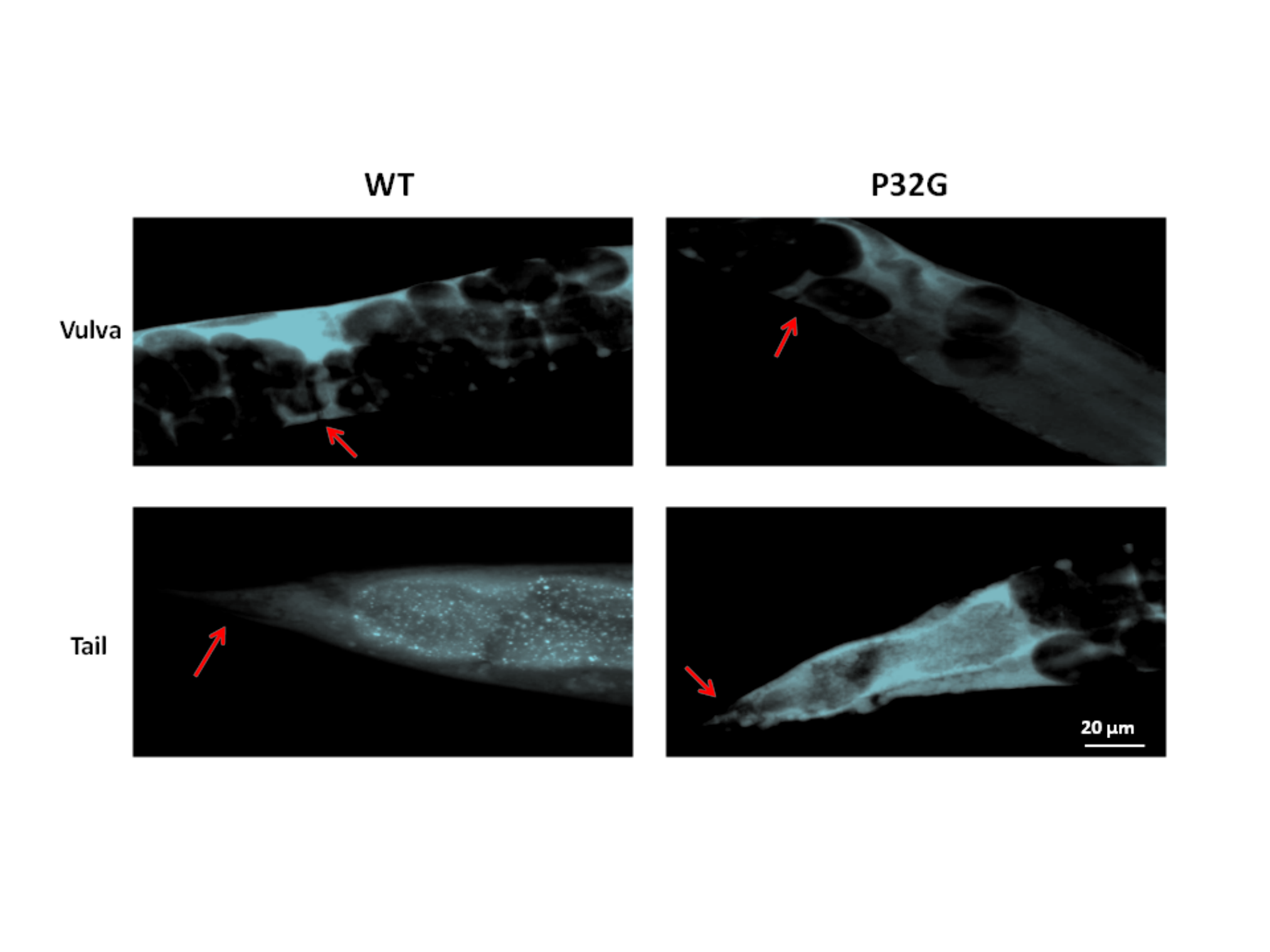

Supplement: Figure S1 — X-34 staining of whole transgenic worms. Representative images of X-34 staining of whole-mount and fixed sections of WT and P32G transgenic worms. Animals depicted are 1–2 day adult worms. X-34 staining was visualized at short wavelength excitation. Red arrows pointed at vulva muscles and anal sphincter muscle in the tail where a specific β2-m related signal was observed with immunofluorescence studies (see Figure 3). The X-34 signal observed was not due to amyloid deposition but to intestine related non-specific background. Scale bar, 20 µm. (TIF) [file pone.0052314.s001.tif]
